# The Moral Stereotypes of Liberals and Conservatives: Exaggeration of Differences across the Political Spectrum

**DOI:** 10.1371/journal.pone.0050092

**Published:** 2012-12-12

**Authors:** Jesse Graham, Brian A. Nosek, Jonathan Haidt

**Affiliations:** 1 Psychology Department, University of Southern California, Los Angeles, California, United States of America; 2 Psychology Depatment, University of Virginia, Charlottesville, Virginia, United States of America; 3 Stern School of Business, New York University, New York, New York, United States of America; Boston College, United States of America

## Abstract

We investigated the moral stereotypes political liberals and conservatives have of themselves and each other. In reality, liberals endorse the individual-focused moral concerns of compassion and fairness more than conservatives do, and conservatives endorse the group-focused moral concerns of ingroup loyalty, respect for authorities and traditions, and physical/spiritual purity more than liberals do. 2,212 U.S. participants filled out the Moral Foundations Questionnaire with their own answers, or as a typical liberal or conservative would answer. Across the political spectrum, moral stereotypes about “typical” liberals and conservatives correctly reflected the direction of actual differences in foundation endorsement but exaggerated the magnitude of these differences. Contrary to common theories of stereotyping, the moral stereotypes were not simple underestimations of the political outgroup's morality. Both liberals and conservatives exaggerated the ideological extremity of moral concerns for the ingroup as well as the outgroup. Liberals were least accurate about both groups.

## Introduction

“The national Democratic Party is immoral to the core. Any American who would vote for Democrats is guilty of fostering the worst kind of degeneracy. The leaders of this party are severely out of touch with mainstream, traditional American values. They are crusaders for perversion, for licentiousness, for nihilism and worse.”

—Joseph Farah [1], *World Net Daily*

“Republicans don't believe in the imagination, partly because so few of them have one, but mostly because it gets in the way of their chosen work, which is to destroy the human race and the planet. Human beings, who have imaginations, can see a recipe for disaster in the making; Republicans, whose goal in life is to profit from disaster and who don't give a hoot about human beings, either can't or won't.”

—Michael Feingold [2], *Village Voice*

For as long as there have been political rivalries there have been unflattering stereotypes painted by each side about the other. These stereotypes go far beyond clichés about latte liberals and gun-rack conservatives; as the quotations above show, they often include the claim that the other side is immoral or downright evil.

Of course, evil is in the eye of the beholder, and liberal and conservative eyes seem to be tuned to different wavelengths of immorality. For conservatives, liberals have an “anything goes” morality that says everything should be permitted for the sake of inclusion and diversity, no matter how bizarre or depraved (e.g., [3]). For liberals, conservatives lack basic moral compassion, especially for oppressed groups, and take a perverse joy in seeing the rich get richer while innocents suffer in poverty (e.g., [4]). These views may be caricatures, but they suggest that accusations of immorality may differ in content depending on the ideologies of the source and the target. In this paper we use Moral Foundations Theory [5] to investigate liberals' and conservatives' *moral stereotypes* of themselves and each other—that is, their expectations about how strongly typical partisans would endorse values related to each of five intuitive moral foundations. Our study was designed to answer three questions: 1. How accurate are these moral stereotypes? 2. Are they exaggerations of real differences in moral values? 3. Where on the political spectrum do we find the greatest accuracy? Rather than examining general beliefs about the immorality of the other side, we sought a finer resolution of the moral domain to provide the first identification of patterns of inaccuracy for moral concerns.

### Exaggeration and Accuracy in Stereotypes

Although the literature on stereotypes has tended to concentrate on biases and inaccuracies, several reviews have noted the accuracy of many social stereotypes in terms of real group differences [6], [7], [8]. The notion that stereotypes could be *exaggerations* of actual group differences was popularized by Allport [9] in *The Nature of Prejudice*: “a stereotype is an exaggerated belief associated with a category” (p.191). Stereotypes have long been thought of as motivated exaggerations both of stereotypical characteristics (Irish people are drunk *every day*) and in overgeneralization (*Every* Irish person is drunk every day).

A review by McCauley [10], however, found only weak support for stereotypes-as-exaggeration as a general cognitive process. For instance, McCauley & Stitt [11] found general accuracy with some *under*estimation of group differences when White students were asked to estimate characteristics of Black students. But in the cases of racial, gender and occupational groups McCauley [10] reviews, there may be motives to appear *un*prejudiced against outgroups, and these motives might counteract exaggeration tendencies. In cases where one does not wish to hide signs of intergroup hostility, motivational factors may have the opposite effect, increasing exaggeration and stereotyping.

This brings us to politics, where people are quite willing to report their preferences for ingroups over outgroups (e.g., [12]), and sometimes even relish the opportunity. Social identity theory [13] has been applied to political partisans, positing a motivation to maximize distinctions between the political ingroup and outgroup based on identifications with one's own political party [14]. Examining the accuracy of stereotypes about the issue positions of Democrats and Republicans, Judd and Park [15] found more exaggeration in the outgroup (vs. ingroup) stereotypes of either side; outgroup stereotype exaggeration was strongest for those most identified with their ingroup, suggesting that partisans of either side exaggerate more than moderates and centrists. Although this work made use of moral issues, we have found no studies looking specifically at the content of *moral* stereotypes, and how such stereotypes might be driven by processes beyond simple partisan outgroup derogation.

### Moral Stereotyping along Five Foundations

Moral Foundations Theory was created to identify the moral content areas most widely discussed in the anthropological and evolutionary literatures. The theory posits five best candidates for being the psychological “foundations” upon which moral virtues and institutions can be socially constructed. The first two foundations are Harm/care (involving intuitions of sympathy, compassion, and nurturance) and Fairness/reciprocity (including notions of rights and justice). These two foundations are generally concerned with the protection and fair treatment of individuals; they are therefore called the two “individualizing” foundations. The other three foundations, in contrast, are called the “binding” foundations because they underlie moral systems in which people are bound into larger groups and institutions. (These labels are not meant to imply that welfare and fairness concerns can never be group-focused, or that the others can never be individual-focused; see [16]). These foundations are Ingroup/loyalty (supporting moral obligations of patriotism and “us vs. them” thinking); Authority/respect (including concerns about traditions and maintaining social order) and Purity/sanctity (including moral disgust and spiritual concerns about treating the body as a temple).

Graham, Haidt, and Nosek [17] found that liberals endorsed the individualizing foundations (Harm, Fairness) more than conservatives did, whereas conservatives endorsed the binding foundations (Ingroup, Authority, Purity) more than liberals did. This pattern has been observed across a variety of samples and methods, including self-report measures of (un)willingness to violate the foundations for money, text analyses of sermons in liberal and conservative churches, content coding of life narratives, and facial muscle movements [18], [19], [20], [21].

If this pattern is found so consistently, are people aware of these differences? Research on partisan stereotypes [15], as well as research on naïve realism and the culture war [22], suggests that the two sides will overestimate their differences on specific issues. Might they likewise exaggerate differences in fundamental moral concerns, stereotyping their opponents as immoral/amoral monsters? Would these moral stereotypes be characterized by general derogation of outgroup morality, or would there be more complexity or asymmetry to the stereotypes?

To examine the moral stereotypes that liberals and conservatives hold about each other, we took advantage of a method introduced by Dawes, Singer, and Lemons [23] of having partisans indicate the values of “typical” partisan group members, allowing comparison of these projections with the partisans' actual answers. Participants completed multiple versions of the Moral Foundations Questionnaire (MFQ; [16]). One version asked participants for their own responses; we refer to these as the “actual” scores. The other two versions asked participants to complete the MFQ as a “typical liberal” would, or as a “typical conservative” would; we refer to these as the “moral stereotype” scores. These versions allow us to assess moral stereotypes about liberals and conservatives, and to quantify their accuracy by comparing them to the responses people gave for themselves.

Regarding our first research question (Are moral stereotypes accurate?), because of the pervasiveness of the actual liberal-conservative differences, we predicted that participants would, on average, correctly guess that liberals value the individualizing foundations more than conservatives do, and that conservatives value the binding foundations more than liberals do. Regarding our second question (Are these stereotypes exaggerations of real group differences?), although McCauley [10] found only weak evidence for a general cognitive process of stereotypes-as-exaggeration, we expected that the hostility between liberals and conservatives could create motivations to exaggerate the existing group differences. It is even possible that liberals and conservatives would exaggerate the moral concerns of their own group, not just the outgroup, perhaps as motivation to further distinguish their group from the other [14]. Regarding our third question (Who is most accurate?) we find reasons in the literature to generate three hypotheses, among which we hoped to adjudicate:

1. *Moderates most accurate*. Studies on ideological polarization (e.g., [24], [25]), the ideological extremity hypothesis [e.g.], [ 26], [27,28], and naïve realism [22] suggest a *symmetrical* exaggeration of differences when liberals and conservatives try to look at the world through the eyes of the other. Partisans should distort equally (presumably by underestimating their opponents' moral concerns) because both sides think the other side does not truly care about morality. On this view, political moderates should be the most accurate, morally stereotyping liberals and conservatives the least.
2. *Liberals most accurate*. Social psychological work on conservatism [see 29, 30 for meta-analytic reviews] shows relations between conservatism or authoritarianism and mental rigidity, intolerance, and close-mindedness. Similarly, Carter et al. [31] found that acceptance of stereotyping was highest in individuals with conservative traits. These findings suggest that conservatives might be more threatened and less able to see the world from an alternate moral standpoint, and therefore more motivated to stereotype liberals than vice-versa.
3. *Conservatives most accurate.* Moral Foundations Theory suggests that liberals may have a harder time understanding conservatives' morality than vice-versa. If liberals don't intuitively feel what could be considered moral about Ingroup (racism?), Authority (oppression?), and Purity (sexual Puritanism?), then they may be forced to conclude that conservatives simply don't care about morality—specifically, that conservatives don't care about Harm and Fairness, because they support policies that seem to hurt and cheat people for no morally good reason.

Following the existing stereotype literature, we consider the first hypothesis to be the default prediction: if the results only show outgroup derogation by partisans about each other, then moral stereotypes are no different than other forms of stereotyping. However, if the results show asymmetrical inaccuracies (hypotheses 2 and 3), inaccuracies about the ingroup as well as the outgroup, or overestimations as well as underestimations of moral values, then this would suggest that moral stereotypes involve novel psychological processes beyond the well-understood intergroup stereotyping processes driving exaggeration of outgroup characteristics.

## Methods

### Participants

The participants were 2,212 visitors (62% female; median age 28; only U.S. residents or citizens) to ProjectImplicit.org, where they were randomly assigned to this study. All participants in the research pool had previously filled out demographic information, including sex, age, and political identity (7-point scale, strongly liberal to strongly conservative). 1,174 participants self-identified using one of the three liberal options, 538 chose the “moderate” midpoint, and 500 chose one of the three conservative options. Data from 77 participants were excluded because of high ratings on the catch item of the MFQ; removal of these participants did not significantly alter any of the results. The study was approved by the University of Virginia Institutional Review Board.

### Materials

The MFQ consists of two parts, moral relevance and moral judgments. In the relevance part, participants indicate the moral relevance of foundation-related concerns (e.g., “whether or not someone did something disgusting,” for Purity) on a 6-point scale, from never relevant to always relevant. In the judgments section, participants rated their agreement with foundation-related statements (e.g., “It is more important to be a team player than to express oneself,” for Ingroup) on a 6-point scale, from strongly disagree to strongly agree. Alphas for the foundation subscales were .71 (Harm), .69 (Fairness), .58 (Ingroup), .73 (Authority), and .83 (Purity).

### Procedure

To keep the study session brief and repetitiveness to a minimum, we capitalized on the power of a large sample with a planned missingness design [32]. Participants were randomly assigned to complete four of six possible questionnaires: 2 (moral relevance or moral judgments), by 3 (answered as oneself, as a “typical liberal”, or as a “typical conservative”). (Results for questionnaires answered as oneself are reported in Graham, et al. [17], Study 2. Participants also completed an Implicit Association Test that is not relevant for this report.) Because participants completed four out of the six possible measures, all of our 2,212 participants completed two to four measures as they thought a typical political partisan would complete them.

Instructions for the liberal [and conservative] versions of the moral relevance measures read as follows:

When A TYPICAL LIBERAL [CONSERVATIVE] decides whether something is right or wrong, to what extent are the following considerations relevant to the liberal's [conservative's] thinking? Remember, instead of selecting your own answers, answer all questions as a typical liberal [conservative].

Instructions for the moral judgments measure read as follows:

Please read the following statements and indicate the extent to which A TYPICAL LIBERAL [CONSERVATIVE] would agree or disagree. Remember, instead of selecting your own answers, answer all questions as a typical liberal [conservative].

### Comparison datasets

To gauge the accuracy of participants' predictions of “typical” liberal and conservative responses, we needed a standard of comparison. The most obvious comparisons were the *actual* ratings provided by the liberals and conservatives in our sample, when they were asked to answer as themselves. This was indeed our first comparison. However, it is not ideal because our sample is not representative of the national population. For instance, our sample of conservatives contains a higher proportion of self-described slight conservatives than a representative population would. We therefore created a second comparison dataset by selecting the actual responses of self-reported extreme liberals and conservatives (the two endpoints of our 7-point politics measure). If the moral stereotypes are equivalent or stronger than these extremes, then they are likely to be exaggerations compared to the average liberal or conservative in the general population. A second reason for including the extreme comparisons is that people may imagine a “typical” liberal/conservative to be a party-line prototype rather than an average partisan, and so accuracy may be better measured in terms of extremes than averages.

To further increase confidence in our exaggeration interpretations, we also obtained scores for a short-form MFQ collected from a nationally-representative sample [33]. This dataset is the result of a random-digit-dialing survey given to 1,001 individuals by Knowledge Networks. The two samples had four items in common for every foundation except Ingroup, which had one item in common. Comparisons between the moral stereotypes and this nationally-representative dataset include only the items common to both datasets.

## Results

We measured and analyzed accuracy at the level of moral foundations subscores, aggregates of 4–5 items each; this allowed us to capture accuracy in terms of mean foundational concerns, as well as relative rankings of the five foundations. For the ten MFQ subscores (five foundations measured by relevance and judgments subscales) we compared predicted (moral stereotype) scores answered as a typical liberal or typical conservative to four criteria: (a) the actual liberal/conservative means in the current sample, (b) the actual means for extreme liberals/conservatives in the current sample, (c) the actual liberal/conservative means in the representative sample, and (d) the actual means for extreme liberals/conservatives in the representative sample.

As an example, the mean Harm-relevance score for all participants answering as a typical conservative was 2.46, with a standard deviation of 1.11 (see Table 1). The actual mean of conservatives in the sample was 3.43 (SD .95), meaning that people on average underestimated how morally relevant conservatives would find Harm concerns, *t*(477.53) = −13.52, *p*<.001, *d* = −1.24. We compared such overall moral stereotype scores (using the entire sample) as well as the moral stereotypes held by liberals, moderates, and conservatives separately. Means for all comparisons (ten foundation subscores answered as a typical liberal and typical conservative, compared to the four comparison criteria) are available in Table 1, and the comparison statistics are available in Table 2 and a supplemental spreadsheet; the spreadsheet calculates *t, df,* and *d* for each comparison (see example above) using formulas that do not assume equal variances or Ns. Below we present meta-analytic summaries of these comparisons. We organize the results around answers to our three central questions.

**Table 1:** Predicted and actual Moral Foundations Questionnaire subscore means and standard deviations.

		Answers as Typical Liberal		Actual Liberal answers				Answers as Typical Conservative		Actual Conservative answers			
				All Liberals		Extreme Liberals				All Conservatives		Extreme Conservatives	
		Mean	SD	Mean	SD	Mean	SD	Mean	SD	Mean	SD	Mean	SD
Relevance:	Harm	3.77	0.93	4.00	0.80	4.11	0.80	2.46	1.11	3.43	0.95	3.23	1.11
	Fairness	3.87	0.90	3.78	0.82	4.00	0.76	2.29	1.07	3.11	0.91	3.11	1.12
	Ingroup	2.53	0.89	2.60	0.94	2.40	0.95	3.12	0.90	2.72	0.98	2.46	1.17
	Authority	2.36	0.86	2.44	0.84	2.26	0.86	3.41	0.84	2.88	0.77	3.04	0.87
	Purity	2.06	1.00	2.33	0.97	2.14	1.02	3.38	0.97	3.02	0.95	3.14	1.21
Judgments:	Harm	3.72	0.92	3.53	0.88	3.64	0.90	2.72	1.10	2.98	0.92	2.92	0.95
	Fairness	3.79	0.87	3.76	0.79	4.00	0.75	2.55	1.02	3.05	0.78	2.90	0.91
	Ingroup	1.81	0.81	1.85	0.88	1.73	0.84	2.96	0.89	2.32	0.81	2.52	0.95
	Authority	2.05	1.09	2.39	0.99	2.05	0.97	4.08	0.94	3.58	0.74	3.93	0.74
	Purity	1.62	1.12	1.63	1.09	1.38	1.13	3.74	1.12	3.04	1.01	3.27	1.03

		Answers as Typical Liberal		Actual Liberal answers				Answers as Typical Conservative		Actual Conservative answers			
				All Liberals		Extreme Liberals				All Conservatives		Extreme Conservatives	
		Mean	SD	Mean	SD	Mean	SD	Mean	SD	Mean	SD	Mean	SD
Relevance:	Harm	3.81	1.03	3.77	0.96	4.31	0.53	2.35	1.22	3.43	1.10	3.57	1.16
	Fairness	3.85	1.03	3.57	0.96	3.92	0.70	2.25	1.22	3.22	1.04	3.35	1.15
	Ingroup	2.36	1.27	2.93	1.23	2.92	1.38	3.12	1.30	3.18	1.33	3.39	1.22
	Authority	2.34	1.13	2.95	0.95	2.48	1.15	3.81	0.98	3.30	1.03	3.54	1.00
	Purity	1.73	1.16	2.53	1.22	2.17	1.40	3.52	1.12	3.16	1.19	3.54	1.13
Judgments:	Harm	3.73	1.15	3.23	1.03	3.50	1.11	2.32	1.38	2.78	1.16	2.84	1.17
	Fairness	4.18	1.01	3.95	0.81	4.13	0.77	3.07	1.30	3.63	0.99	3.41	1.17
	Authority	2.39	1.27	3.40	0.96	2.96	1.17	4.26	0.98	4.00	0.79	4.11	0.95
	Purity	1.72	1.38	2.71	1.18	2.10	1.41	3.64	1.21	3.53	0.98	4.01	0.95

*Note.* Top panel shows predicted and actual answers for the study sample, and bottom panel shows the same for the comparison to the nationally-representative dataset, using only items common to both datasets (no items in common for Ingroup judgments). Sample sizes for each statistic, as well as predicted “typical” answers broken down by liberals, moderates, and conservatives, can be found in the supplement.

**Table 2:** Statistical comparisons of actual and predicted answers for liberals, moderates, and conservatives.

		Typical Liberal answers compared to sample average:									Typical Conservative answers compared to sample average:								
		Lib			Mod			Con			Lib			Mod			Con		
		*t*	*df*	*d*	*t*	*df*	*d*	*t*	*df*	*d*	*t*	*df*	*d*	*t*	*df*	*d*	*t*	*df*	*d*
Relevance:	Harm	−1.89	1281.15	−0.11	−6.33	427.36	−0.61	−6.47	385.75	−0.66	−17.93	571.72	−1.50	−9.08	532.89	−0.79	−5.12	535.73	−0.44
	Fairness	5.22	1270.77	0.29	−2.65	435.51	−0.25	0.14	418.73	0.01	−15.99	580.92	−1.33	−7.44	526.33	−0.65	−3.81	532.36	−0.33
	Ingroup	−3.44	1253.77	−0.19	1.22	520.94	0.11	0.49	481.33	0.04	7.72	469.87	0.71	5.12	546.99	0.44	1.88	543.74	0.16
	Authority	−1.91	1264.17	−0.11	1.09	464.44	0.10	−3.85	441.87	−0.37	9.82	572.81	0.82	7.41	527.65	0.65	6.35	541.44	0.55
	Purity	−4.72	1278.15	−0.26	−0.77	491.63	−0.07	−7.33	451.77	−0.69	6.53	531.09	0.57	2.30	539.96	0.20	3.74	545.00	0.32
Judgments:	Harm	6.51	1286.48	0.36	0.45	462.67	0.04	1.28	475.18	0.12	−5.16	626.68	−0.41	−0.90	524.02	−0.08	−1.25	505.71	−0.11
	Fairness	6.04	1279.04	0.34	−3.74	428.03	−0.36	−3.73	481.93	−0.34	−9.37	700.27	−0.71	−4.47	512.56	−0.39	−3.67	503.77	−0.33
	Ingroup	−3.79	1270.49	−0.21	1.52	576.13	0.13	2.06	551.54	0.18	12.13	580.37	1.01	7.59	529.00	0.66	5.83	521.19	0.51
	Authority	−8.53	1284.70	−0.48	−0.35	445.33	−0.03	−4.96	448.14	−0.47	8.66	670.83	0.67	4.77	475.30	0.44	7.31	523.64	0.64
	Purity	−3.87	1278.19	−0.22	4.74	494.39	0.43	1.10	468.42	0.10	10.51	580.14	0.87	4.47	509.34	0.40	7.99	523.15	0.70

		Typical Liberal answers compared to sample extreme:									Typical Conservative answers compared to sample extreme:								
		Lib			Mod			Con			Lib			Mod			Con		
		*t*	*df*	*d*	*t*	*df*	*d*	*t*	*df*	*d*	*t*	*df*	*d*	*t*	*df*	*d*	*t*	*df*	*d*
Relevance:	Harm	−3.01	326.80	−0.33	−5.78	478.58	−0.53	−5.87	461.51	−0.55	−6.04	36.17	−2.01	−2.93	41.84	−0.91	−1.20	40.97	−0.38
	Fairness	0.11	339.27	0.01	−4.62	475.86	−0.42	−2.60	460.96	−0.24	−6.37	35.97	−2.12	−3.21	41.19	−1.00	−1.68	40.26	−0.53
	Ingroup	0.37	304.77	0.04	3.19	434.43	0.31	2.59	433.84	0.25	4.96	34.93	1.68	3.80	38.78	1.22	2.36	38.40	0.76
	Authority	1.50	308.12	0.17	2.95	456.37	0.28	−0.76	447.19	−0.07	2.85	36.41	0.95	2.27	42.60	0.69	1.82	40.53	0.57
	Purity	−0.83	316.98	−0.09	1.39	440.46	0.13	−3.59	440.19	−0.34	1.99	35.28	0.67	0.44	39.54	0.14	1.08	38.00	0.35
Judgments:	Harm	2.96	352.95	0.31	−0.96	484.70	−0.09	−0.35	482.81	−0.03	−1.99	55.74	−0.53	−0.09	67.70	−0.02	−0.27	67.93	−0.07
	Fairness	0.21	371.54	0.02	−5.85	491.58	−0.53	−6.25	490.83	−0.56	−3.24	55.83	−0.87	−1.22	64.72	−0.30	−0.82	63.53	−0.21
	Ingroup	−0.89	352.20	−0.09	2.90	451.04	0.27	3.28	464.37	0.30	4.15	52.35	1.15	2.51	60.93	0.64	1.62	59.53	0.42
	Authority	−1.62	377.07	−0.17	3.17	493.39	0.29	−0.31	494.19	−0.03	1.50	58.22	0.39	0.14	82.66	0.03	1.08	65.07	0.27
	Purity	0.34	338.81	0.04	6.06	471.20	0.56	3.17	483.64	0.29	3.53	54.63	0.96	1.06	72.24	0.25	3.02	58.37	0.79

		Typical Liberal answers compared to representative sample average:									Typical Conservative answers compared to representative sample average:								
		Lib			Mod			Con			Lib			Mod			Con		
		*t*	*df*	*d*	*t*	*df*	*d*	*t*	*df*	*d*	*t*	*df*	*d*	*t*	*df*	*d*	*t*	*df*	*d*
Relevance:	Harm	2.46	391.36	0.25	−1.08	515.34	−0.09	−1.45	488.15	−0.13	−17.92	680.00	−1.37	−8.80	550.21	−0.75	−6.56	560.31	−0.55
	Fairness	6.63	401.81	0.66	0.07	509.87	0.01	1.31	495.63	0.12	−17.88	699.29	−1.35	−8.48	533.44	−0.73	−3.78	538.53	−0.33
	Ingroup	−7.85	402.02	−0.78	−3.28	509.00	−0.29	−3.97	493.61	−0.36	0.33	639.17	0.03	−0.43	571.93	−0.04	−2.58	570.16	−0.22
	Authority	−7.30	461.20	−0.68	−4.64	506.78	−0.41	−9.26	488.12	−0.84	8.02	638.68	0.63	4.96	579.52	0.41	6.54	589.20	0.54
	Purity	−10.03	394.04	−1.01	−4.40	500.56	−0.39	−9.09	495.01	−0.82	6.73	619.98	0.54	1.83	576.95	0.15	1.90	584.48	0.16
Judgments:	Harm	8.37	435.83	0.80	3.38	512.00	0.30	2.95	513.68	0.26	−7.08	778.47	−0.51	−1.72	523.85	−0.15	−3.12	510.30	−0.28
	Fairness	6.64	440.90	0.63	0.48	491.21	0.04	−0.31	503.19	−0.03	−9.24	827.57	−0.64	−3.21	502.35	−0.29	−3.56	468.95	−0.33
	Authority	−12.91	523.46	−1.13	−7.01	494.97	−0.63	−10.06	498.34	−0.90	4.69	804.21	0.33	2.34	473.33	0.22	3.85	523.87	0.34
	Purity	−12.84	462.81	−1.19	−4.84	512.59	−0.43	−7.61	515.71	−0.67	2.78	797.42	0.20	−1.58	475.99	−0.14	1.50	534.26	0.13

		Typical Liberal answers compared to representative sample extreme:									Typical Conservative answers compared to representative sample extreme:								
		Lib			Mod			Con			Lib			Mod			Con		
		*t*	*df*	*d*	*t*	*df*	*d*	*t*	*df*	*d*	*t*	*df*	*d*	*t*	*df*	*d*	*t*	*df*	*d*
Relevance: Harm	−2.17	30.64	−0.78	−2.79	51.19	−0.78	−2.85	55.95	−0.76	−7.91	40.32	−2.49	−4.55	47.82	−1.32	−3.68	46.42	−1.08
	Fairness	0.59	28.32	0.22	−1.45	40.00	−0.46	−1.02	38.80	−0.33	−7.78	40.17	−2.46	−4.33	47.47	−1.26	−2.31	46.83	−0.67
	Ingroup	−2.96	26.41	−1.15	−1.33	29.51	−0.49	−1.58	30.78	−0.57	−0.85	42.26	−0.26	−1.12	50.26	−0.32	−2.18	49.95	−0.62
	Authority	−0.46	26.68	−0.18	0.08	30.42	0.03	−1.76	30.62	−0.64	1.89	41.58	0.59	1.01	48.08	0.29	1.79	46.24	0.53
	Purity	−2.24	26.27	−0.88	−0.44	28.53	−0.16	−2.48	28.84	−0.92	0.77	41.46	0.24	−1.00	48.96	−0.29	−1.05	47.34	−0.31
Judgments: Harm	1.87	26.89	0.72	0.26	30.71	0.09	0.14	31.68	0.05	−3.00	43.69	−0.91	−1.03	51.88	−0.29	−1.71	51.36	−0.48
	Fairness	1.43	27.50	0.54	−0.59	36.67	−0.20	−0.92	35.66	−0.31	−2.50	42.79	−0.76	−0.34	49.49	−0.10	−0.52	50.72	−0.15
	Authority	−2.81	27.16	−1.08	−1.07	31.59	−0.38	−2.24	31.74	−0.80	1.13	42.01	0.35	0.37	51.13	0.10	1.02	46.02	0.30
	Purity	−2.38	26.72	−0.92	0.14	30.10	0.05	−0.93	29.83	−0.34	−1.28	44.84	−0.38	−2.85	59.59	−0.74	−2.05	50.30	−0.58

*Note.* Top panel compares predicted and actual answers for the study sample, and bottom panel compares predictions to actual answers in the nationally-representative dataset, using only items common to both datasets (no items in common for Ingroup judgments). Lib = Liberal participants, Mod = Moderate participants, Con = Conservative participants. Formulas used to calculate *t, df,* and *d* without assuming equal sample sizes or variances can be found in the supplement.

*1. Are the moral stereotypes accurate with regard to the direction of liberal-conservative differences in the foundations? Yes*. For both relevance and judgment items, answers as a typical liberal yielded higher scores on Harm and Fairness than answers as a typical conservative (*t*s>23.83, *p*s<.001, *d*s>1.00), and lower scores on Ingroup, Authority, and Purity (*t*s<−15.76, *p*s<.001, *d*s>0.65). These showed directional accuracy compared to the real group differences found both in this study (see below) and in previous research: liberals endorse individual-focused moral concerns more than conservatives do, and conservatives endorse group-focused moral concerns more than liberals do.

**Figure 1:**
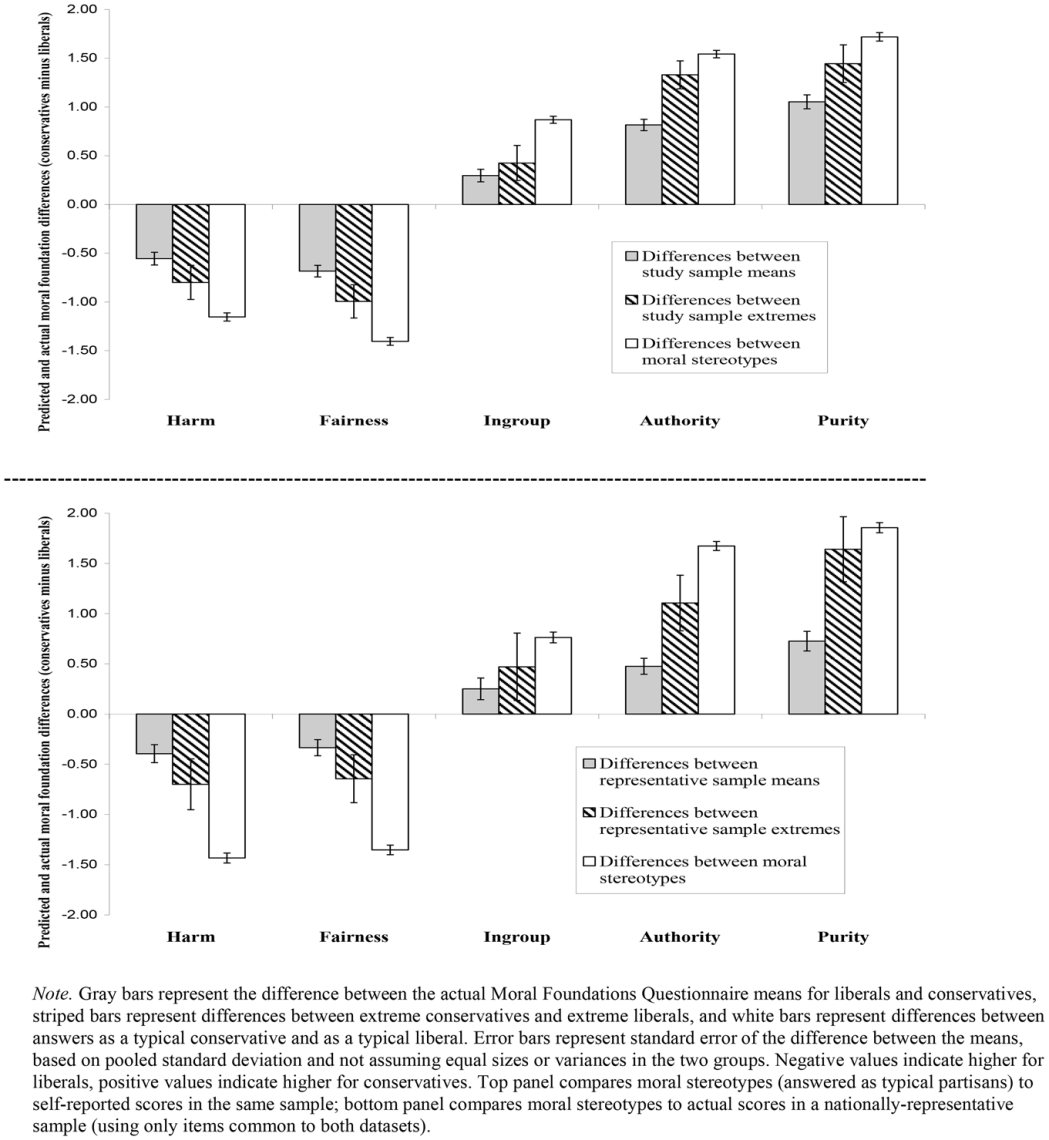
Comparisons of moral stereotypes to actual conservative-liberal differences in moral foundation endorsement.

*2. Are these stereotypes exaggerations of the real group differences? Yes*. Figure 1 shows the average conservative-liberal differences for each foundation, comparing the moral stereotypes (answered as typical partisans) to the actual differences found in our four comparison criteria (current sample means, current sample extremes, representative sample means, representative sample extremes). For all of the measures, foundation differences were similar across formats (relevance and judgments), and so for clarity of presentation the two MFQ subscales are combined in Figure 1. Differences were calculated as follows: the overall moral foundation means for answered-as-typical-liberal versions were subtracted from the overall means for the same scores answered as a typical conservative. Differences between the actual means of liberals and conservatives, and between the actual means of extreme liberals and extreme conservatives, were calculated the same way (see Table 3).

**Table 3:** Actual conservative-liberal differences compared to those predicted by liberals, moderates, and conservatives.

		Actual differences (con - lib):				Predicted differences (Typical con - Typical lib)							
		Sample average		Sample extremes		Everyone		Lib		Mod		Con	
		Difference	S.E.	Difference	S.E.	Difference	S.E.	Difference	S.E.	Difference	S.E.	Difference	S.E.
Relevance:	Harm	−0.57	0.07	−0.88	0.20	−1.31	0.04	−1.78	0.05	−0.97	0.09	−0.58	0.09
	Fairness	−0.67	0.06	−0.89	0.20	−1.57	0.04	−2.02	0.05	−1.14	0.09	−0.99	0.09
	Ingroup	0.12	0.07	0.06	0.21	0.58	0.04	0.78	0.05	0.46	0.08	0.24	0.08
	Authority	0.43	0.06	0.77	0.16	1.05	0.04	1.09	0.05	0.90	0.08	1.11	0.08
	Purity	0.69	0.07	0.99	0.22	1.32	0.04	1.39	0.05	0.95	0.09	1.53	0.09
Judgments:	Harm	−0.55	0.07	−0.72	0.15	−1.00	0.04	−1.25	0.06	−0.65	0.09	−0.74	0.09
	Fairness	−0.70	0.06	−1.10	0.14	−1.24	0.04	−1.63	0.05	−0.80	0.08	−0.75	0.08
	Ingroup	0.47	0.06	0.79	0.15	1.15	0.04	1.41	0.05	0.93	0.07	0.76	0.07
	Authority	1.20	0.06	1.89	0.13	2.04	0.04	2.22	0.05	1.59	0.10	2.04	0.09
	Purity	1.41	0.07	1.89	0.17	2.12	0.05	2.47	0.06	1.47	0.10	1.97	0.09

		Actual differences (con - lib):				Predicted differences (Typical con - Typical lib)							
		Sample average		Sample extremes		Everyone		Lib		Mod		Con	
		Difference	S.E.	Difference	S.E.	Difference	S.E.	Difference	S.E.	Difference	S.E.	Difference	S.E.
Relevance:	Harm	−0.34	0.09	−0.74	0.22	−1.45	0.05	−1.89	0.06	−1.08	0.10	−0.81	0.10
	Fairness	−0.35	0.08	−0.57	0.23	−1.60	0.05	−2.14	0.06	−1.14	0.10	−0.82	0.10
	Ingroup	0.25	0.11	0.47	0.34	0.76	0.05	0.98	0.07	0.57	0.11	0.45	0.12
	Authority	0.35	0.08	1.06	0.28	1.48	0.04	1.47	0.06	1.22	0.10	1.78	0.09
	Purity	0.63	0.10	1.37	0.33	1.79	0.05	2.01	0.06	1.28	0.10	1.80	0.10
Judgments:	Harm	−0.45	0.09	−0.66	0.29	−1.42	0.05	−1.75	0.07	−0.96	0.11	−1.07	0.11
	Fairness	−0.32	0.08	−0.72	0.24	−1.11	0.05	−1.50	0.06	−0.66	0.10	−0.63	0.10
	Authority	0.60	0.08	1.15	0.28	1.87	0.05	2.00	0.06	1.51	0.10	1.92	0.10
	Purity	0.82	0.09	1.91	0.32	1.92	0.05	2.26	0.07	1.24	0.12	1.82	0.11

*Note.* Top panel shows predicted and actual answers for the study sample, and bottom panel shows the same for the comparison to the nationally-representative dataset, using only items common to both datasets (no items in common for Ingroup judgments). S.E. = standard error of the difference between the means, based on pooled standard deviations and not assuming equal sizes or variances in the two groups. Lib = Liberal predictors, Mod = Moderate predictors, Con = Conservative predictors.

As both the top panel (comparisons to current sample) and bottom panel (comparisons to representative sample) of Figure 1 show, moral stereotypes exaggerated the liberal-conservative differences in line with Moral Foundations Theory. Not only were the moral stereotypes about typical partisans more different from each other (average absolute difference 1.41, range 0.58–2.12) than the actual MFQ scores of liberals and conservatives (average absolute difference = 0.57, range 0.12–1.41), they were as different or even moreso than the actual scores of extreme partisans (average absolute difference = 0.98, range 0.06–1.91; see Figure 1 and Table 3). That is, participants' beliefs about the “typical” liberal and conservative were even more polarized than the actual polarization between *extreme* liberals and conservatives.

*3. Who is most accurate? It depends on the type of morality.* Comparisons to actual group means were also made separately for the moral stereotypes held by liberals, moderates, and conservatives. This allows us to address our third research question about who is most accurate when answering as a typical liberal or typical conservative. Statistics and effect sizes for each of these comparisons (the three groups' moral stereotypes about typical liberals and conservatives compared to the four actual group criteria, for five foundations, gauged by relevance and judgments measures) were calculated (see Table 2). Here we meta-analytically summarize the comparisons using ranges and averages of effect sizes, gauging accuracy in terms of differences from the current sample means and (using only items common to both datasets) the representative sample means.

*3a. Conservatives were most accurate about the individual-focused moral concerns of either side, and liberals were least accurate.* Compared to actual group means of either data set, moral stereotypes about the typical conservative showed substantial underestimation of conservatives' Harm and Fairness concerns. Liberals tended to underestimate the most (average *d* = −.98, −1.50≤*d*s≤−.41), followed by moderates (average *d* = −.48, −.79≤*d*s≤−.08); conservatives underestimated the individualizing concerns of the typical conservative the least (average *d* = −.34, −.55≤*d*s≤−.11), but they too underestimated their own group's Harm and Fairness concerns in every comparison with actual conservative scores.

Stereotypes about the Harm and Fairness concerns of the typical liberal tended to be more accurate as compared to actual liberal scores in the two datasets. Here again conservatives were the most accurate, only slightly underestimating liberal individualizing concerns (average *d* = −.08, −.66≤*d*s≤.26), followed by moderates, who underestimated slightly more (average *d* = −.12, −.61≤*d*s≤.30). Liberals were the least accurate about their own group's individualizing concerns, *over*estimating them on average (average *d* = .40, −.11≤*d*s≤.80).

*3b. Moderates were most accurate about the group-focused moral concerns of either side, and liberals were least accurate.* Stereotypes about the Ingroup, Authority, and Purity concerns of the typical conservative tended to be overestimations compared to the actual group means in both datasets. Here again liberals were the least accurate, overestimating conservative binding concerns the most (average *d* = .55, .03≤*d*s≤1.01), followed by conservatives, who also overestimated their own group's binding concerns (average *d* = .34, −.22≤*d*s≤.70); moderates were the most accurate (average *d* = .28, −.14≤*d*s≤.66), but they too overestimated the binding concerns when answering as a typical conservative.

Stereotypes about the typical liberal, on the other hand, tended to underestimate the binding moral concerns actual liberals reported. Here again liberals were the least accurate, underestimating their own binding concerns the most (average *d* = −.62, −1.19≤*d*s≤−.11), followed by conservatives (average *d* = −.46, −.90≤*d*s≤.18). Moderates were the most accurate (average *d* = −.17, −.63≤*d*s≤.43), but also underestimated the binding concerns when answering as a typical liberal.

**Figure 2:**
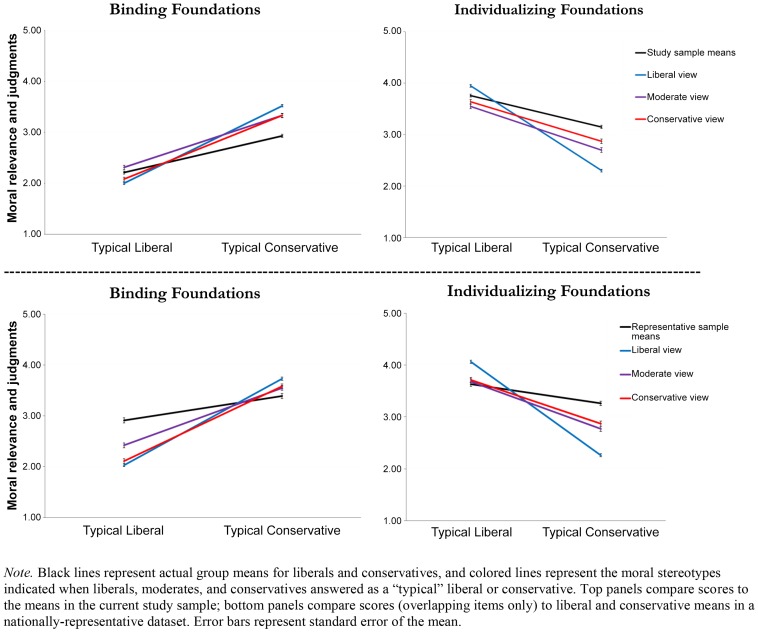
Moral stereotypes about the typical liberal's and typical conservative's endorsement of the binding foundations (Ingroup, Authority, Purity), and individualizing foundations (Harm, Fairness).

**Figure 3:**
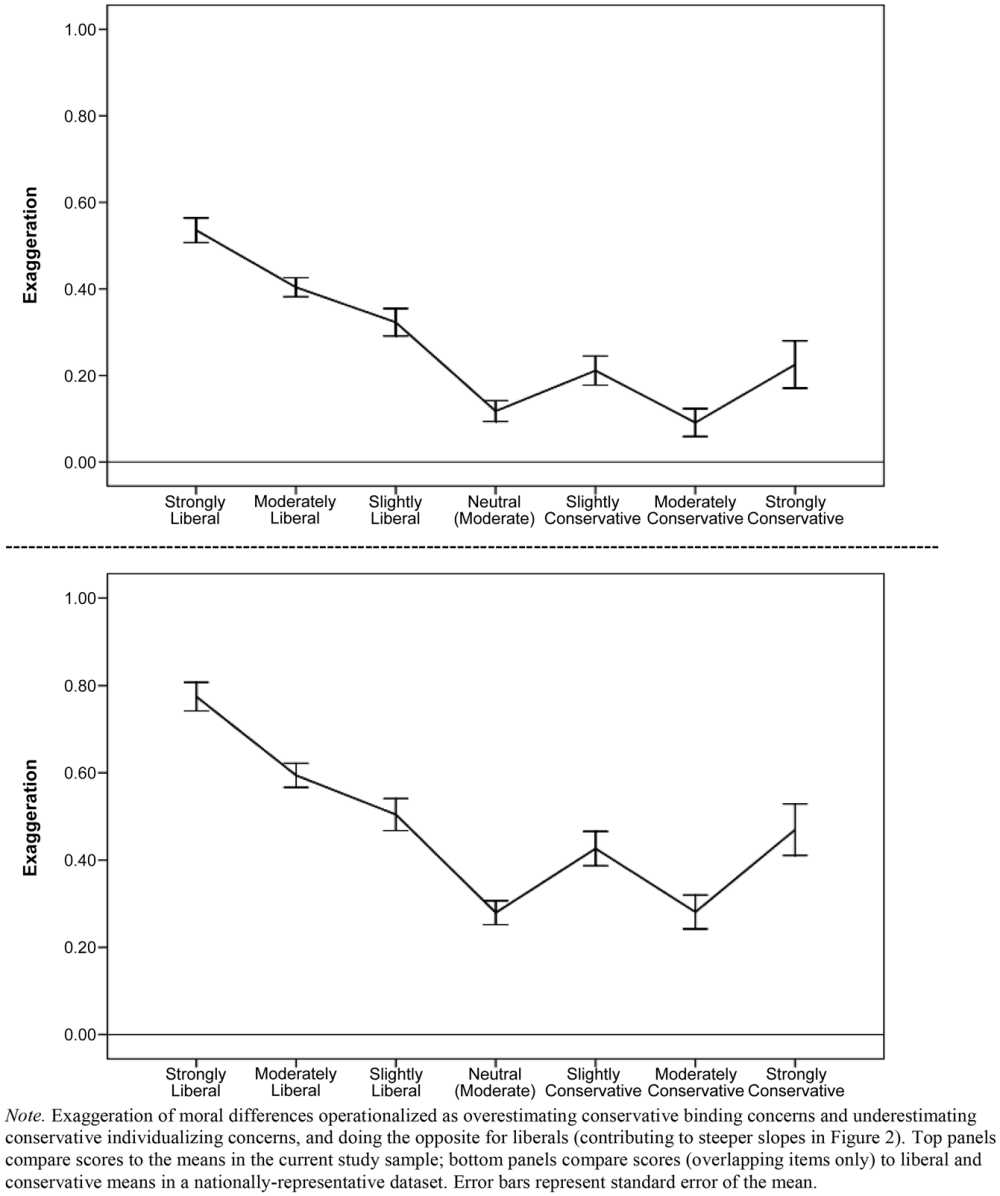
Exaggeration of moral differences across political ideology.

*3c. Liberals exaggerate moral differences the most.* Means for the three groups' moral stereotypes about the typical liberal and typical conservative are shown compared to the real group means (solid black lines) in Figure 2. As both of the top panels (current sample comparison) and both of the bottom panels (representative sample comparison) show, participants across the political spectrum tended to exaggerate the liberal-conservative differences, as evidenced by the steeper slopes of the prediction lines as compared to the actual lines. This exaggeration of differences is an effect of overestimating liberals' individualizing concerns and underestimating their binding concerns, and overestimating conservatives' binding concerns and underestimating their individualizing concerns. All four panels of Figure 2 show that liberals exaggerate differences the most (lines representing moral stereotypes held by liberals have the steepest slopes); the figure also shows that the largest inaccuracies were liberal underestimations of the individualizing concerns of the typical conservative. Overall exaggeration of moral differences (operationalized as overestimating conservative binding concerns, underestimating conservative individualizing concerns, and doing the opposite for liberals) is plotted across the full ideological spectrum in Figure 3.

## Discussion

Results indicate that people at all points on the political spectrum are at least intuitively aware of the actual differences in moral concerns between liberals and conservatives: they correctly predicted that liberals would care more than conservatives about the two individualizing foundations and that conservatives would care more than liberals about the three binding foundations. The results also confirm previous studies of partisan misperception [24] by showing that, in general, people overestimate how dramatically liberals and conservatives differ. Remarkably, people even morally stereotype their own ingroup, with liberals overestimating liberals' strong individualizing concerns and underestimating liberals' weak binding concerns, and conservatives exaggerating conservatives' moral concerns in the opposite directions.

Our results go beyond previous studies, however, in finding and explaining an otherwise puzzling result: liberals were the least accurate. We presented three competing hypotheses about accuracy: 1) We found some support for the hypothesis that moderates would be most accurate, which they were in the case of the binding foundations. However, and most crucially, partisan inaccuracies were not mirror images of each other (in which case the red and blue lines in Figure 2 would have opposite slopes). On the contrary, liberals and conservatives both tended to exaggerate their binding foundation differences by underestimating the typical liberal and overestimating the typical conservative. 2) We found no support for the hypothesis that liberals would be most accurate; liberals were the least accurate about conservatives *and* about liberals. The largest inaccuracies were in liberals' underestimations of conservatives' Harm and Fairness concerns, and liberals further exaggerated the political differences by overestimating their own such concerns. 3) Finally, we found some support for the hypothesis that conservatives would be the most accurate, which they were in the case of the individualizing foundations. In line with Moral Foundations Theory, liberals dramatically underestimated the Harm and Fairness concerns of conservatives. These findings add to the literature on moral foundations by demonstrating a novel form of pragmatic validity [16] for the theory: conceptualizing and measuring the moral stereotypes people have of different social groups.

While we obtained a nationally-representative sample for comparison of MFQ scores, it is important to note that the predicted answers as typical liberals/conservatives all came from a non-representative Project Implicit sample. However, the participants in this study do “run the gamut” across the ideological spectrum, from very liberal to very conservative, and Figure 3 demonstrates exaggeration across all 7 points on the political orientation item. Extreme liberals exaggerated the moral political differences the most, and moderate conservatives did so the least. Further, Nosek, Banaji, and Jost [34] showed evidence that strong conservatives at Project Implicit preferred conservative candidates, both implicitly and explicitly, as much as strong liberals preferred liberal candidates. Finally, across Project Implicit studies the liberal and conservative extremes show equivalent or near-equivalent extremity in implicit and explicit liking and identity with partisan parties, politicians, and positions [35], [36].

Nevertheless, we cannot completely rule out reference effects in these predictions based on non-representative sampling. In particular, while the conservatives in this sample are indeed conservative, they may also have different social experiences than a representative conservative. For example, conservatives who live in urban or predominately liberal enclaves might have greater insight into liberal beliefs than conservatives who live in rural or predominately conservative enclaves. A useful follow-up investigation would examine the effect of exposure to liberals and conservatives in one's social context. If this is impactful, and if the present sample is systematically skewed in this regard, then accounting for social context may qualify the present conclusion of conservatives having greater accuracy than liberals. It is also worth noting that our single ideology item did not allow for participants to indicate that they were libertarian, or that they were liberal on social issues but conservative on economic issues. Research on libertarians has revealed a pattern of moral concerns unlike that of liberals, moderates, or conservatives [37]; this finding, along with their coherent ideological identity separate from liberals and conservatives, makes libertarians a particularly interesting sample for future studies using this paradigm. Do libertarians share the moral stereotypes about liberals and conservatives shown by participants in the current study? Do people hold consistent moral stereotypes about the “typical” libertarian, and are they accurate? Besides addressing these questions, future work should investigate different possible antecedents of moral stereotyping, such as differential exposure to ideological caricatures in the media.

The ideological “culture war” in the U.S. is, in part, an honest disagreement about ends (moral values that each side wants to advance), as well as an honest disagreement about means (laws and policies) to advance those ends. But our findings suggest that there is an additional process at work: partisans on each side exaggerate the degree to which the other side pursues moral ends that are different from their own. Much of this exaggeration comes from each side underestimating the degree to which the other side shares its own values. But some of it comes, unexpectedly, from overestimating the degree to which “typical” members of one's own side endorse its values. Studies of ingroup stereotypes tend to show that they are more accurate and less exaggerated than stereotypes about an outgroup [38], especially for higher-status groups like Whites [39]. However, the current study found that moral stereotypes about an ideological group can be just as exaggerated when held by ingroup members as by outgroup members, and sometimes even more so. We suspect that this is partially due to the fact that one can imagine members of one's own ideological group more extreme than oneself; people could in fact be motivated to differentiate themselves from their ideological group, imagining “typical” group members to be more extreme in their moral profile (it would be interesting in future work to obtain measures of how “typical” participants rate themselves to be – perhaps everyone likes to see themselves as atypical when it comes to politics). But this may also be a unique feature of moral stereotypes, in that people are motivated to exaggerate the moral values of their group in ways that are in line with those same values.

The asymmetrical pattern found in moral stereotypes about the individualizing foundations fits remarkably well with recent work on ideological opponent and own-group misperceptions. Examining co-perceptions of conflicting groups such as pro-life/pro-choice and hawks/doves, Chambers and Melnyk [40] found that partisans saw their adversaries as motivated by an opposition to their own core values, rather than being motivated by promotion of the adversaries' values. This is consistent with the moral stereotypes that liberals appear to have of conservatives: liberals see conservatives as being motivated by an opposition to liberals' core values of compassion and fairness, as well as being motivated by their own (non-moral) values of ingroup loyalty, respect for authorities and traditions, and spiritual purity (they may be particularly likely to focus on issues in which these values come into conflict). This misperception is asymmetrical: conservatives did underestimate liberal moral concerns with the binding foundations, but they were no more likely to underestimate than liberals themselves.

It is striking that instead of basic partisan outgroup derogation, in which both sides predict that the other is less moral in general, we found foundation-specific moral stereotypes about liberals and conservatives—and these moral stereotypes were largely shared by all. Participants across the political spectrum exaggerated liberal moral disregard for Ingroup, Authority, and Purity, and conservative disregard for Harm and Fairness—that is, exaggerations of the patterns predicted by Moral Foundations Theory. This suggests that moral stereotypes might be unique in that they are motivated (partisans want to cast the other side as immoral) and yet partisans share the *same* moral stereotypes about either side. Even more surprising, they share both of these moral stereotypes with moderates, who are presumably not as motivated to stereotype either side. More research is needed to further delineate the moral stereotypes of political partisans, for instance to see if moral stereotypes about members of political parties mirror those about ideological groups, both in two-party political systems like the U.S. and in multiparty systems like Italy. We also hope that future studies can use Moral Foundations Theory's finer resolution of the moral domain to investigate specific moral stereotypes along other social groupings, such as race, gender, social class, age, or weight.

Chambers and Melnyk [40] conclude: “Partisan group members suffer the misapprehension that their adversaries work to actively and willfully oppose their own sides' interests rather than promoting the values that are central to their adversaries' doctrine…it is this perception that may spawn the feelings of distrust and animosity that partisans feel toward their rivals and may ultimately fuel conflict between partisan groups” (p.1309). In this study, we focused on the moral values of ideological opponents, and their perceptions of the moral values of either side, in order to understand the moral “distrust and animosity” endemic to the liberal-conservative culture war. We found that there are real moral differences between liberals and conservatives, but people across the political spectrum exaggerate the magnitude of these differences and in so doing create opposing moral stereotypes that are shared by all. Calling attention to this unique form of stereotyping, and to the fact that liberal and conservative moral values are less polarized than most people think, could be effective ways of reducing the distrust and animosity of current ideological divisions.
